# Text Messaging: An Intervention to Increase Physical Activity among African American Participants in a Faith-Based, Competitive Weight Loss Program

**DOI:** 10.3390/ijerph14040326

**Published:** 2017-03-29

**Authors:** Pamela McCoy, Sophia Leggett, Azad Bhuiyan, David Brown, Patricia Frye, Bryman Williams

**Affiliations:** 1 School of Public Health, Jackson State University, Jackson, MS 39217, USA; sophia.s.leggett@jsums.edu (S.L.); azad.r.bhuiyan@jsums.edu (A.B.); patricia.a.frye@jsums.edu (P.F.); 2 College of Medicine, University of Central Florida, Orlando, FL 32827, USA; dmb2029@gmail.com; 3 College of Liberal Arts, Jackson State University, Jackson, MS 39217, USA; bryman.e.williams@jsums.edu

**Keywords:** text messaging, African Americans, public health, health disparity, health education, physical activity, obesity, health communication, health behavior, behavioral theory

## Abstract

African American adults are less likely to meet the recommended physical activity guidelines for aerobic and muscle-strengthening activity than Caucasian adults. The purpose of this study was to assess whether a text message intervention would increase physical activity in this population. This pilot study used a pre-/post-questionnaire non-randomized design. Participants in a faith-based weight loss competition who agreed to participate in the text messaging were assigned to the intervention group (*n* = 52). Participants who declined to participate in the intervention, but agreed to participate in the study, were assigned to the control group (*n* = 30). The text messages provided strategies for increasing physical activity and were based on constructs of the Health Belief Model and the Information-Motivation-Behavioral Skills Model. Chi square tests determined the intervention group participants increased exercise time by approximately eight percent (*p* = 0.03), while the control group’s exercise time remained constant. The intervention group increased walking and running. The control group increased running. Most participants indicated that the health text messages were effective. The results of this pilot study suggest that text messaging may be an effective method for providing options for motivating individuals to increase physical activity.

## 1. Introduction

In recent years, the United States has experienced a dramatic increase in obesity rates among its citizens. According to the World Health Organization, overweight and obesity are defined as abnormal or excessive fat accumulation that may impair health [1]. The Centers for Disease Control and Prevention define overweight or obesity as weight that is higher than what is considered as a healthy weight for a given height. Body Mass Index, or BMI, is used as a screening tool for overweight or obesity. Body Mass Index (BMI) is a person’s weight in kilograms divided by the square of height in meters. A high BMI can be an indicator of high body fatness. An adult who has a BMI between 25 and 30 is considered overweight and an adult who has a BMI of 30 or higher is considered obese. A BMI equal to or greater than 40 is classified as extreme or severe obesity [2].

These rates are continuously rising to epidemic levels and threatening the health and lives of millions of Americans. People who identified as black alone or black in combination with one or more other races accounted for 14% of the U.S. Population in 2010 [3]. This racial/ethnic group is disproportionately affected by obesity, and therefore, by diseases for which obesity is a risk factor. Additionally, African Americans are less likely to be physically active than other groups.

Racial/ethnic and gender disparities in obesity prevalence exist among U.S. adults. In 2011–2012, African Americans had the highest obesity prevalence among all racial/ethnic groups at 47.8%. Among men, non-Hispanic black men had a 37.1% prevalence of obesity, surpassed only by Hispanic men with 40.1%. Non-Hispanic black women fared worse than all other women with an obesity prevalence of 56.6% [4].

Of 24 states with an obesity prevalence of 35% or greater, five states have an African American population of 25% or more. Those states are: Alabama, Georgia, Louisiana, Mississippi, and South Carolina [5,6].

Obesity is a risk factor for heart disease, cancer, stroke, diabetes, and kidney disease, all of which are among the top 10 leading causes of death for African Americans in 2014 [7]. People who engage in physical activity are at a lower risk for heart disease, stroke, type 2 diabetes and some cancers. Physical activity can also aid in controlling weight [8]. Only 18% of non-Hispanic black adults compared to 23% of non-Hispanic white adults meet the 2008 Physical Activity Guidelines for aerobic and muscle-strengthening activity. Women (46%) are less likely to meet guidelines for aerobic activity than men (54%). Residents of some southern states are less physically active than people living in other regions of the country [9].

States with the highest African American population and the highest obesity prevalence are among those with between 26.7% and 36% of their adult citizens who report they did not participate in any leisure-time physical activities during the past month [10].

Adherence to increasing physical activity and maintaining a healthy body weight, as well as good nutrition, can decrease the risk of developing serious health conditions and can help manage these conditions so that they do not get worse over time [11].

An easily available and cost-effective health communication, behavioral change intervention such as text messaging that is acceptable for promoting physical activity to a large population is needed.

One purpose of this study was to assess whether text messages to communicate health information via mobile telephones would promote an increase in physical activity, and therefore, weight loss among study participants as compared to participants who did not receive text messages. Secondly, this study sought to assess the effect text messages had on the knowledge, beliefs and behaviors of the intervention participants regarding physical activity options and weight loss.

The use of text messages as an intervention is particularly significant because it addresses the Healthy People (HP) 2020 health communication and health information technology main goal to “use health communication strategies and health information technology (IT) to improve population health outcomes and health care quality and to achieve health equity”. HP 2020 also indicates that communication, information and technology that people interact with on a daily basis shape their ideas about health and behavior as well as the manner in which they search for, understand and use health information, significantly impacting their decisions and actions about their health [12].

According to Healthy People 2020, ensuring that all Americans …… participate in regular physical activity, and achieve and maintain a healthy body weight is critical to improving the health of Americans at every age [11]. Thus, a specific health promotion issue is developing and determining a viable health communication behavioral change intervention that is acceptable, easily available, and cost-effective, such as text messaging, to promote physical activity that will improve healthful behaviors for a large population. Therefore, the purpose of this study was to assess whether health promotion using text messages to communicate health information via mobile telephones would increase physical activity and weight loss among the participants.

The need for inclusion of different age groups, settings, contexts, and geographic locations was a gap in text messaging studies commonly recommended by the review authors of 15 systematic reviews and/or meta-analyses representing 89 relevant individual studies. The review, conducted by Hall, Cole-Lewis and Bernhardt, focused on text message interventions targeting adults [13].

This study addresses this gap by utilizing an intervention population of overweight and obese African American adults with a mean age of 52.75 years, a racial/ethnic and age group which is most susceptible to overweight and obesity. The obesity rate for adults age 45 to 64 in Mississippi is between 35% and 40%, according to Behavioral Risk Factor Surveillance System statistics for 2015 [14].

The setting for this study was a faith-based community participating in a weight loss competition in Mississippi. To the authors’ knowledge, no studies have addressed the gap of the feasibility of utilizing text messaging as a health promotion tool with the demographics of this population within the context of a faith-based weight loss competition in the geographic location of a southern state.

This study aimed to utilize text messaging as a health communication tool that could address several Healthy People 2020 objectives, including: providing personalized self-management tools and resources, delivering accurate, accessible, and actionable health information that is targeted or tailored, increasing health literacy skills, providing new opportunities to connect with culturally-diverse and hard-to-reach populations, and providing sound principles in the design of programs and interventions that result in healthier behaviors [12].

Text messaging is widely used among the American public; and therefore, may be advantageous as a behavior change intervention. Text messaging interventions have been utilized as an effective behavior change tool in prevention trials for a variety of health issues. Studies using text messaging have shown promise in improving health outcomes such as weight loss [15] smoking cessation [16], and chronic disease care [17,18].

According to Cole-Lewis and Kershaw, few studies in a review of 17 articles representing 12 studies that provided an overview of behavior change interventions for disease management and prevention delivered through text messaging specified a theoretical rationale [19]. The authors indicated those text message interventions designed and measured using behavioral theory are more likely to be successful. To address the theoretical gap, this study utilized a behavioral theory (the Health Belief Model (HBM)), and a communication theory (the Information-Motivation-Behavioral (IMB) Skills Model), to design the intervention and guide development of the text messages. None of the studies found in the literature utilized both the HBM and IMB [20].

## 2. Materials and Methods

### 2.1. Design

The research design for this pilot study was quasi-experimental. Specifically, the research design was a nonrandomized, control group pre-test–post-test design [21]. A mixed methodology was used to collect data from a convenience sample. Quantitative data were collected via written, self-administered pre- and post-questionnaires. The questionnaires were developed using the demographic and exercise (physical activity) sections of the 2011 Behavioral Risk Factor Surveillance System (BRFSS) Questionnaire [22]. Qualitative data were collected via an unstructured interview process [23].

### 2.2. Participants

The target population for this text messaging study consisted of participants who attended primarily urban churches located mainly in central Mississippi and were enrolled in a ten-month predominantly African American faith community weight loss competition. The sample for this study was a convenience sample of adults, age 21 and older who volunteered to participate in the three-month study.

Organized religious membership is a part of many African American’s lives making it conducive to conducting health promoting activities. 

Campbell et al. stated, “Churches also provide an attractive venue to recruit and retain participants, given that they tend to be stable institutions with members who attend frequently over many years” [24].

### 2.3. Participant Recruitment

Study participants were recruited by church team leaders and/or the researcher. Church team leaders who were willing to serve as recruiters for participants from their churches attended an informational and training session about the study conducted by the researcher. The session included a project overview, goals and objectives, the role and responsibilities of team leaders and information on human subjects’ protection. Team leaders were asked to complete a human subjects training and provide their certificate of completion to the researcher. Recruitment packets were prepared and distributed to church team leaders following the training session.

Participants signed a consent form and agreed to complete a pre- and post-questionnaire. The intervention group was comprised of members of the target population who agreed to participate in the health text messages component of the study. The control group was comprised of weight loss program participants who did not want to participate in the health text messages component of the study. Each participating church in the intervention group and the control group designed their own specific customized weight loss program. This study incorporated the text message intervention into the existing weight loss programs at the participating intervention churches.

No incentives were provided to the study participants. The protocol for this study was approved by the Jackson State University Institutional Review Board.

### 2.4. Formative Research

Designing the text messaging intervention began with formative research conducted via unstructured interviews with key informants who were demographically representative of the target population. The unstructured interviews allowed key informants to assist in developing the text messages by providing tips and strategies about physical activity they would find beneficial in helping them increase their physical activity.

Unstructured interviews were conducted with members of nine churches. The nine churches were representative of all denominations in the study. The unstructured interviews with key informants included questions about the type and frequency of text messages needed. Based on key informants’ requests, no more than three messages should be sent per week; and messages should be sent on appropriate days and at appropriate times when participants would be more likely to find them helpful. Key informants recommended that no text messages be sent on weekends. Text messages sent on Fridays would be beneficial for the weekend. 

There was no consensus among key informants on the time of day that messages should be sent; therefore, the researcher developed the timeframe for sending text messages; for example, messages encouraging physical activity were sent close to morning or afternoon work break times; and physical activity messages suggesting housework or yard work were sent on Fridays, when participants may have more time to perform those tasks.

### 2.5. Intervention

The theoretical framework for the physical activity health text messaging intervention combined aspects of the Health Belief Model (HBM) and the Information-Motivation-Behavioral (IMB) Skills model, because both use communication as a tool for influencing behavior [20].

The intervention was developed to offer text messages designed to motivate participants to increase their physical activity. The text messages were based on one or more of the theoretical models’ constructs: (1) cues to action; and (2) self-efficacy for the HBM; and (3) acquisition of relevant and useful information and facilitation of skill development for the IMB Skills Model.

Text messages based on the HBM cues to action construct included a verb recommending an action for the participant to perform. For example, “Park at the far end of the parking lot and walk to the store entrance. Walk down every aisle of the grocery store”. 

The IMB construct of acquisition of relevant and useful information was accomplished by providing information about ways to increase physical activity and the benefits. An example is “High intensity activities require less time than low intensity activities to burn the same amount of calories. Go higher! Race walk, jog, run, swim, cycle!”

Relevant and useful information coupled with the cues to action were aimed at facilitating the development of appropriate or new skills, all of which aimed to enhance participants’ self-efficacy.

The text messages, created by the researcher, were based on or adapted from information found on publically available Web sites of reliable health-related governmental agencies and private and public non-profit organizations. Participants received three messages per week either in the morning or afternoon on alternate days of alternate weeks. Some messages were sent on appropriate days and at appropriate times of the day when participants would be more likely to find them helpful. Messages encouraging physical activity were sent close to morning or afternoon work break times; and messages suggesting housework or yard work were sent on Fridays. No messages were sent on Saturdays or Sundays, except the introductory message which was sent the weekend prior to the beginning of the intervention. The final text message served to motivate participants to sustain their weight loss.

### 2.6. Data Collection

The data collection process was accomplished in two phases, once at the beginning of the study and again at the end of the study. Pre- and post-questionnaires consisted of two sections. Questions in the first section included demographics, church affiliation, height and weight, and physical activity habits. The organization sponsoring the weight-loss competition measured participants’ pre- and post-weights using a professional digital scale identified by number. Participants entered their weights on the questionnaires. In section two, if the participants indicated that they were willing to participate in the health messages intervention, they were asked to complete questions designed to facilitate delivery of the health messages via a text message on their cell phones. An answer of “no” to the question regarding willingness to receive text messages served as a disqualifier for the intervention study group and as a qualifier for the control group.

The post-questionnaire was identical to the pre-questionnaire with the exception of some previously reported demographic questions. The post-questionnaire also included questions about the frequency of reading the health text messages, preference for receipt of health text messages and open-ended questions about what participants liked and disliked about the health text messages and changes they would recommend.

A Likert scale was used to assess the participants’ views about the impact of the health text messages on their knowledge of physical activity options, on helping them believe that they could increase their physical activity, and whether the health text messages helped them increase their physical activity.

### 2.7. Statistical Analyses

Quantitative data were analyzed using the Statistical Package for the Social Sciences (SPSS, Chicago, IL, USA), Version 22. Descriptive statistics were used to analyze demographic characteristics and Likert scale results regarding the effect health text messages had on knowledge, beliefs and behaviors of the intervention group participants about physical activity options. Cronbach’s alpha was 0.89.

An independent samples *t*-test compared the mean weight loss of the intervention group participants and the control group participants. McNemar’s test was used to determine frequencies of physical activity participation by intervention and control group participants. Results were considered to be statistically significant if *p* < 0.05.

Qualitative data were analyzed by identifying and coding the emerging themes from open-ended questions assessing what the intervention group participants liked and disliked about the health text messages and their suggestions for changes.

## 3. Results

The study included a total of 82 participants with a mean age of 52.83 years and a mean body weight of 204.13 pounds all of whom completed a pre-questionnaire and post-questionnaire. Participants were African American, 87.8% female, 12.2% male, married (52%) and had obtained a post-graduate degree (42.7%). The average annual household income was $50,001 and above (47.3%). Demographic characteristics of the intervention and control groups are shown in Table 1.

The religious faiths reported by participants indicated that a majority of the sample was protestant (90%) with the remainder reporting a non-denominational church affiliation (10%). 

### 3.1. Physical Activity

Two items used to assess whether intervention group participants who received health text messages would increase their physical activity more than participants in the control group who did not receive health text messages were: (1) the length of time (minutes or hours) of participation in physical activity; and (2) an increase in the type of physical activity done most often, i.e., walking, cycling, jumping rope or running (Table 2 and Table 3).

Comparing the change in the length of time of physical activity participation (minutes/hours) in the intervention and control groups, (Table 2) the intervention group increased in participating in physical activity for less than 30 m from 4% (*n* = 2) at pre-intervention to 12% (*n* = 6) at post intervention. There was a decrease in physical activity participation of 30 m to more than two hours for the intervention group from 96% (*n* = 48) to 88% (*n* = 44). The *p*-value was statistically significant (*p* = 0.03).

The control group remained constant in physical activity participation for less than 30 m at 14.8% (*n* = 4) and in participating in physical activity for 30 m to more than two hours at 85% (*n* = 23) from pre-test to post-test without intervention during the study period. The *p*-value (*p* = 1.0) was not statistically significant.

Comparing the change in the type of physical activity done most often in the intervention and control groups (Table 3), the intervention group increased walking from 78.8% (*n* = 41) to 80.8% (*n* = 42) from pre-intervention to post intervention and increased running from 9.6% (*n* = 5) to 15.4% (*n* = 8) from pre-intervention to post-intervention. The control group increased in running from 16.7% (*n* = 5) to 23.3% (*n* = 7) from pre-questionnaire to post-questionnaire without intervention during the study period. The *p*-values for the types of physical activity done most often were not statistically significant.

### 3.2. Text Messages Effect

The effect that health text messages had on knowledge, beliefs and behaviors of the intervention group participants regarding physical activity options was assessed by asking participants to respond, “strongly agree”, “agree”, “neutral”, “disagree” or “strongly disagree” to a seven-item Likert scale about their perception of the health text messages effectiveness on the following: (1) The health text messages increased my knowledge about physical activity options; (2) The health text messages helped me believe that I could increase my physical activity; and (3) The health text messages helped me toward my weight loss goal.

Overall, most participants indicated that the health text messages were effective. As shown in Table 4, the health text messages that received the highest percentages of “strongly agree” responses were those that helped participants believe that they could increase their physical activity (39.2%), and those that increased their knowledge about physical activity options (39.2%). Two percent of participants disagreed that the health text messages helped increase physical activity. Approximately one third of participants (33.3%) strongly agreed that the health text messages helped toward their weight loss goal, and 5.9% disagreed with the statement. No participants “strongly disagreed” with any of the health text message statements.

## 4. Discussion

Studies using text messaging as a behavioral intervention varied on many aspects of the intervention, including number and frequency of text messages sent, time frame of study, use of an additional Internet/Web-based component, and one-way or two-way text communication.

Number and frequency of text messages sent varied widely with the majority being sent once per week, followed by three times per week. The most text messages sent were five times per day. Time frames for the studies ranged from six weeks to one year, three months. The majority of studies had a time frame of either three months or one year and longer. The shortest studies were conducted in six weeks.

Approximately one-third of the studies did not incorporate an Internet or Web-based component. The majority of studies utilized one-way text message communication to the participants.

The weight loss outcomes and acceptability of the text messaging intervention resulting from this pilot study are similar to other studies in the review of literature.

In this study, it was found that a modestly greater weight loss occurred among intervention group participants compared to the control group participants. Although not statistically significant, this achievement of more weight loss in the intervention group was consistent with findings from text message interventional studies that provided information on diet, exercise and/or behavior modification conducted by Joo and Kim [25], Kim et al. [26], Haapala et al. [27], Patrick et al. [28], and Lubans et al. [29]. Additionally, this pilot study was similar to the Kim et al. [26] study, in which body weight of the intervention group was not significantly different from the control group, but differed from pre-test to post-test. In contrast to this pilot study, Shapiro et al. [15] reported that text messaging had no effect on weight.

In contrast to this pilot study, a study by Newton et al. [18] did not support the use of text messaging to increase physical activity. The authors reported that weekly motivational text messages sent to diabetic adolescents reminding them to wear a pedometer and be active did not increase physical activity nor increase wearing a pedometer.

There were several lessons learned regarding the text messaging intervention. The time of day that participants preferred to receive text messages was mornings (49%), followed by afternoons (33.3%). Two percent preferred both morning and afternoons, while 13.7% indicated no preference and two percent would have preferred to receive messages upon returning home after work.

The number of times participants preferred to receive text messages was one to two times weekly (57.7%), followed by three times weekly (23.1%). Eleven and one-half percent of participants preferred to receive text messages four to five times weekly and 7.7% indicated a preference for receiving text messages six or more times weekly.

The days of the week participants preferred to receive text messages were: Monday (74.5%), Friday (53.2%), Wednesday (51.1%), Thursday (40.4%), Tuesday and Saturday (36.2%), and Sunday (25.5%).

A research gap filled by this study is that it demonstrated the ability of text messaging to promote more frequent and longer duration of physical activity in residents of a state with one of the nation’s highest prevalence of obesity. A further implication of this study is that it demonstrated the usefulness of a text messaging intervention for weight loss with a target population of overweight and obese African American adults with a mean age of 52.75 years, a racial/ethnic and age group which is most susceptible to overweight and obesity. To the knowledge of the researcher, this is the first research study utilizing health communication via text messaging to promote weight loss undertaken with a primarily African American adult population of participants in a faith-based community weight loss competition taking place in this geographic location.

Future implications include long-term, larger scale studies using text messaging solely to improve weight loss through increased physical activity in adults who are not participating in another organized weight loss program should be undertaken to isolate intervention effects and better ascertain its benefits. Studies should target not only African American adults, but other racial/ethnic minority, gender and age group populations with or at risk for high rates of obesity. Multiple measures of weight throughout the study period are needed to further determine the usefulness of this intervention. Future studies could also randomize participants to intervention and control groups from among those already participating in a weight loss program to control for self-selection bias. Furthermore, studies to determine the optimal frequency and time of messages are needed. To enhance weight loss, future studies could also include periodic tracking of weight loss with tailored feedback provided via text message. Measurements of weight loss might also include waist circumference and body fat percentage.

Improvement in weight coupled with the favorable satisfaction of the text messages by a majority of participants suggests there is a need to continue future studies to investigate the utility and value of text-message interventions for weight loss by increased physical activity. 

Future studies should focus on racial/ethnic, gender and age groups that are most disproportionately affected by overweight and obesity. 

Finally, the results of this study should be of interest to public health policymakers as text messaging has the potential to reach and be accepted by a large proportion of the population affected by overweight and obesity. This health communication method can become part of a comprehensive plan to combat obesity by improving healthier food consumption and increasing physical activity. Text messaging is indeed accessible, affordable and relevant to a wide variety of target populations. Policymakers should seek to continue assessing the behavioral and environmental barriers to a healthy lifestyle inherent in the everyday lives of its citizenry and as a result develop and implement comprehensive population-level policies statewide to reduce the effects of the epidemic of obesity, which is now, itself, designated as a ‘disease’ and the associated diseases to which it contributes.

A singular text messaging intervention to communicate options for increasing physical activity participation was found to be satisfactory among most participants in this pilot study’s intervention group. Participants strongly agreed or agreed that the text messages increased their knowledge about physical activity options. The self-efficacy of participants was improved as a majority of participants believed the text messages helped increase their physical activity and helped them toward their weight loss goal. These findings are consistent with studies by Joo and Kim [25], Gerber et al. [30], Haapala et al. [27] and Shapiro et al. [15].

### Study Limitations

There are several limitations to this study. Participants were volunteers of a single racial/ethnic group, mostly female and from one region of a southern state in the United States. There was no attempt to ensure that the intervention and control groups were comparable and there was the potential for self-selection bias. The intervention effect may have been moderate due to participants being in a weight loss program already in progress and being allowed to employ any methods they desired to assist with their weight loss.

Data collection was via a self-administered questionnaire which constituted self-report subject to recall bias and/or social desirability bias. Perhaps a more detailed questionnaire could have been used to gather more specific and comprehensive data on the type, intensity, frequency and duration of physical activity undertaken by participants in a variety of settings.

The small study sample size is likely to have been underpowered to detect statistically significant differences and generalizability is limited.

## 5. Conclusions

The results of this pilot study suggest that text messaging via cellular telephone may be an effective motivational, informative health communication method for providing physical activity options for this population in an effort to enhance weight loss. Strengths of this study are that the sample is a racial/ethnic and gender group residing in a geographic location disproportionately affected by overweight and obesity. Overall benefits to participants were: weight loss, an improvement in their behavioral self-efficacy to increase participation in physical activity, and an increase in their knowledge of options for increased physical activity.

There were few barriers to the utilization of this relatively simple, inexpensive health communication method of text messaging for reaching individuals with behavioral change information options and motivational messages.

This pilot study demonstrated the feasibility and acceptability of using a text messaging intervention to promote physical activity among African American adults in the southeastern region of the United States, a population disproportionately affected by overweight and obesity. This study indicated that, as a result of receiving the text messages, participants increased their knowledge about physical activity options (39.2% strongly agreed and 39.2% agreed), believed that they could increase their physical activity (39.2% strongly agreed, 41.2% agreed), and were helped toward their weight loss goal (33.3% strongly agreed and 35.3% agreed).

## Figures and Tables

**Table 1:** Demographic characteristics.

Variable	*N* = 82		*p*
	Intervention Group	Control Group	
	*n* = 52	*n* = 30	
Race *n* (%)			
African American	52 (100%)	30 (100%)	
Gender *n* (%)			0.347
Male	5 (9.6%)	5 (16.7%)	
Female	47 (90.4%)	25 (83.3%)	
Age (years) *M* (SD)	52.75 (±10.94)	52.97 (±12.62)	0.935
Marital status *n* (%)			0.779
Single	13 (27.1%)	5 (18.5%)	
Married	23 (47.9%)	16 (59.3%)	
Divorced	8 (16.7%)	5 (18.5%)	
Widowed	3 (6.3%)	1 (3.7%)	
Separated	1 (2.1%)	-	
Educational level *n* (%)			0.640
High school diploma/GED	6 (11.5%)	2 (6.7%)	
1+ Years college	8 (15.4%)	2 (6.7%)	
College degree	15 (28.8%)	12 (40%)	
Post grad degree	22 (42.3%)	13 (43.3%)	
Vo-tech school	1 (1.9%)	1 (3.3%)	
Annual household income *n* (%)			0.273
≤$10,000	0 (0%)	2 (7.4%)	
$10,001–$20,000	4 (8.5%)	2 (7.4%)	
$20,001–$30,000	4 (8.5%)	2 (7.4%)	
$30,001–$40,000	13 (27.7%)	3 (11.1%)	
$40,001–$50,000	6 (12.8%)	3 (11.1%)	
≥$50,001	20 (42.6%)	15 (55.6%)	

Note: Due to missing data, counts do not total 82. *M* refers to the mean or average age of the participants. GED is an acronym for General Educational Development. It is also referred to as General Education Diploma.

**Table 2:** Length of time (minutes or hours) of participating in physical activity.

Variable	*n* = 50		*p*	*n* = 27		*p*
	Intervention Group			Control Group		
Physical activity time	Pre	Post	0.03	Pre	Post	1.0
Less than 30 m	2 (4.0)	6 (12.0)		0 4 (14.8)	0 4 (14.8)	
30 m to more than 2 h	48 (96.0)	44 (88.0)		23 (85.0)	0 23 (85.0)	

Note: Due to missing data, counts do not total 82; *n* = 82.

**Table 3:** Type of physical activity done most often.

Variable	Intervention Group		*p*	Control Group		*p*
Physical Activity	Pre	Post		Pre	Post	
Walking	41 (78.8)	42 (80.8)	NS	26 (86.7)	25 (83.3)	NS
Cycling	4 (7.8)	2 (3.9)	NS	3 (10.0)	1 (3.3)	NS
Jumping rope	2 (3.8)	1 (1.9)	NS	1 (3.3)	-	NS
Running	5 (9.6)	8 (15.4)	NS	5 (16.7)	7 (23.3)	NS

Note: Due to missing data, counts do not total 82; *n* = 82. NS stands for non- significant and means that the results were not statistically significant.

**Table 4:** What effect will the health text messages have on knowledge, beliefs and behaviors of intervention group participants regarding physical activity options, and thus their weight loss goal?

Likert Scale Item	Intervention Group *n* = 52			
	Strongly Agree (%)	Agree (%)	Neutral (%)	Disagree (%)
1. The health text messages increased my knowledge about physical activity.	20 (39.2)	20 (39.2)	11 (21.6)	-
2. The health text messages helped me believe that I could increase my physical activity.	20 (39.2)	21 (41.2)	10 (19.6)	-
3. The health text messages helped me increase my physical activity.	14 (28.6)	16 (32.7)	18 (36.7)	1 (2.0)
4. The health text messages helped me toward my weight loss goal.	17 (33.3)	18 (35.3)	13 (25.5)	3 (5.9)

Note: Due to missing data, counts do not total 52.
